# Supplementary material for: WordCommentsAnalyzer: A windows software tool for qualitative research
Source: F1000Res. 2018 Sep 4;7:536. Originally published 2018 May 3. [Version 2] doi: 10.12688/f1000research.14819.2 (PMC6137411; doi:10.12688/f1000research.14819.2)
Supplement: Supplementary file 3 [file f1000research-7-17614-s0002.tgz › b2a79496-4ada-47c4-9682-a99ce867836d.zip!/Eight Iranian Health Journals/Journal of Knowledge and Health_15.docx]

© Journal of Knowledge and Health. This work is licensed under CC BY 4.0 (https://creativecommons.org/licenses/by/4.0/).

Volume & Issue: دوره 9، شماره 3: 1393

Title: نقش طرحواره‌های هیجانی در تاب‌آوری" افراد مبتلا‌به HIV"

http://knh.shmu.ac.ir/index.php/site/article/view/391

مقدمه: ابتلا به عفونت HIV همه ابعاد زندگی اجتماعی، اقتصادی، بهداشت و سلامت روان فرد را تحت تأثير قرار می‌دهد. افراد واکنش‌های متفاوتی به این وضعیت نشان می‌دهند. پژوهش حاضر با هدف بررسی رابطه طرحواره‌های هیجانی با میزان تاب‌آوری و مقاومت افراد مبتلا‌به عفونت HIV انجام شد. مواد و روش‌ها: شرکت‌کنندگان پژوهش ۱۳۰ نفر از بیماران مبتلا‌به عفونت HIV بودند که به مرکز مشاوره بیماری‌های رفتاری بیمارستان امام خمینی (ره) مراجعه نموده بودند و با روش نمونه‌گیری در دسترس انتخاب شدند. داده‌های پژوهش با استفاده از پرسشنامه‌هاي تاب‌آوری کانر و دیویدسون، طرحواره هیجانی لیهی و جمعیت شناختی گردآوری و از طریق روش همبستگی و تحلیل رگرسیون گام به گام مورد بررسی قرار گرفت. نتایج: نتایج رگرسیون گام به گام نشان داد که چهار طرحواره هیجانی: نشخوار فکری، تأییدطلبی از دیگران، دیدگاه ساده‌انگارانه و پذیرش هیجانات ۳۸ درصد میزان تاب‌آوری را (38/0R2=) به شیوه معنادار پیش‌بینی می‌کنند (05/0>P). همچنین براساس تحلیل همبستگی بین طرحواره هیجانی مثبت مانند ابراز احساسات، دریافت تأیید از دیگران، دیدگاه ساده‌انگارانه، ارزش‌های والاتر، توافق و پذیرش احساسات و تاب‌آوری رابطه مثبت معنادار و بین طرحواره هیجانی منفی مانند نشخوار فکری با تاب‌آوری رابطه منفی وجود داشت. نتیجه‌گیری: یافته‌های پژوهش نشان داد طرحواره هیجانی نقش مهمی در میزان تاب‌آوری افراد مبتلا‌به عفونت HIV دارد. لذا، طراحي مداخلات روان‌شناختی در اين زمينه توأم با شناسایی و تغییر طرحواره‌های منفی می‌توانند به بهبود تاب‌آوری افراد کمک کنند.

Title: ارزیابی پلی‌مورفیسم 49AG ژن رمزکننده CTLA-4 در بیماران مبتلا‌به لوپوس اریتماتوی سیستمیک: مطالعه مرور منظم و متاآنالیز

http://knh.shmu.ac.ir/index.php/site/article/view/60

مقدمه: آنتی ژن 4 وابسته به لنفوسیت T سیتوتوکسیک نقش مهمی در بازدارندگی فعالیت سلول‌های T بر عهده دارد. به‌نظر می‌رسد پلی‌مورفیسم 49AG مرتبط با بیماری لوپوس اریتماتوی سیستمیک باشد اما نتایج مطالعاتی که تاکنون انجام شده است متناقض می‌باشد. بنابراین مطالعه حاضر با هدف بررسی بیشتر این ارتباط انجام شده است. مواد و روش‌ها: جستجو از بانک‌های SID (Scientific Information Database)، Iran medex، Irandoc، OVID، Science Direct، Pubmed، Scopus انجام شد. کلیه مقالات تا تاریخ 30 دسامبر 2011 مورد بررسی قرار گرفتند. داده‌ها با استفاده از نرم‌افزار STATA نسخه 11 مورد تجزیه‌و‌تحلیل قرار گرفت. جهت بررسی ارتباط بین داده‌ها، نسبت شانس (OR) و فاصله اطمینان (CI) 95% در نظر گرفته شد. در صورت وجود هتروژنیتی از مدل تصادفی (با استفاده از روش درسیمونیان و لیرد) و در صورت عدم‌وجود هتروژنیتی، از مدل ثابت (با استفاده از روش مانتل هنزل) استفاده گردید. نتایج: در مجموع 15 مطالعه در ارتباط با پلی‌مورفیسم 49AG و بیماری لوپوس اریتماتوی سیستمیک وارد مطالعه گردید. 1705 فرد بیمار و 2299 فرد سالم به‌عنوان گروه مورد بررسی قرار گرفتند. در بررسی حاضر هیچ‌گونه ارتباط معناداری بین پلی‌مورفیسم مورد بررسی و بیماری لوپوس اریتماتوی سیستمیک در هیچ یک از گروه‌های نژادی یافت نشد. نتیجه‌گیری: در متا آنالیز حاضر، هیچ‌گونه ارتباطی بین پلی‌مورفیسم 49AG و بیماری لوپوس اریتماتوی سیستمیک یافت نشد. پیشنهاد می‌گردد در آینده با افزایش حجم نمونه، نتایج دقیق‌تری به‌دست آورد.

Title: بررسی ارتباط سطوح سرمی آدیپونکتین با پروفایل لیپید، تستوسترون و کورتیزول در مردان کم‌تحرک

http://knh.shmu.ac.ir/index.php/site/article/view/390

مقدمه: یکی از آدیپوسایتوکین‌هایی که توسط بافت چربی ترشح می‌گردد، آدیپونکتین است که با چاقی، افزایش مقاومت انسولینی، دیس لیپیدمیا و دیابت کاهش می‌یابد. هدف از مطالعه حاضر بررسی ارتباط سطوح سرمی آدیپونکتین با پروفایل لیپید، تستوسترون و کورتیزول در مردان کم تحرک بود. مواد و روش‌ها: در یک مطالعه نیمه‌تجربی 45 مرد جوان که طی 6 ماه گذشته هیچ‌گونه فعالیت بدنی منظمی نداشتند، به‌عنوان آزمودنی انتخاب شدند. پس از 12 ساعت ناشتایی (در ساعت 8 صبح)، نمونه‌های خون جهت تعیین سطوح شاخص‌هاي خونی شامل آدیپونکتین، پروفایل لیپید، تستوسترون و کورتیزول سرم جمع‌آوری و ویژگی‌های عمومی آزمودنی‌ها نیز مورد سنجش قرار گرفت. جهت تحلیل داده‌های خام از نرم‌افزار آماری 16-SPSS و ضریب همبستگی پیرسون در سطح معنی‌داری 01/0>P استفاده گردید. نتايج: سطوح آدیپونکتین به‌طور معنی‌داری با LDL-C (008/0=P، 25/0-=r)، تری‌گلیسیرید (003/0=P، 30/0-=r) و تستوسترون (002/0=P، 41/0-=r) همبستگی منفی، و با HDL-C (005/0=P، 26/0=r) آزمودنی‌ها همبستگی مثبت داشت. هیچ‌گونه رابطه معنی‌داری بین سطوح آدیپونکتین سرم با کلسترول تام (053/0=P، 21/0-=r) و کورتیزول (089/0= P، 16/0=r) مشاهده نشد. نتیجه‌گیری: به‌نظر می‌رسد در افراد کم تحرک پایین بودن غلظت آدیپونکتین ممکن است با اختلال در کنترل پروفایل لیپید همراه باشد، که این احتمال ابتلا به سندروم متابولیکی و بیماری‌های قلبی عروقی را در این افراد بالا می‌برد. همچنین، با وجود ارتباط مستقیم بین غلظت آدیپونکتین و تستوسترون سرم، ارتباط معني‌داري بین سطوح آدیپونکتین با غلظت کورتیزول سرم در اين مطالعه مشاهده نگرديد.

Title: راهبردهاي سازگاري در بيماران مبتلا‌به سندروم تخمدان پلي‌كيستيك؛ يك مطالعه كيفي

http://knh.shmu.ac.ir/index.php/site/article/view/378

مقدمه: سندروم تخمدان پلي‌كيستيك (PCOS)، آثار عمده‌اي بر ابعاد مختلف زندگي بيماران مبتلا دارد. مفهوم توانايي جهت مقابله با بيماري، مي‌تواند در چگونگي ارزيابي افراد از كيفيت زندگيشان حائز اهميت باشد. بنابراين، درك عميق چگونگي سازش اين بيماران ضروري به‌نظر مي‌رسد. هدف از پژوهش حاضر، فراهم نمودن درك عميق راهبردهاي سازگاري استفاده شده در بيماران مبتلا‌به PCOS مي‌باشد. مواد و روش‌ها: اين مطالعه با رويكرد كيفي و از نوع تحليل محتوي است. جهت انجام آن، از مصاحبه عميق با 20 بيمار مبتلا‌به PCOS، كه به‌صورت مبتني برهدف انتخاب شدند استفاده گرديد. نتايج: درون مايه‌هاي اصلي به‌دست آمده از داده‌ها شامل تراوشات فكري (اعتقادات مذهبي، پارادوكس افكار)، گام‌هاي عملي (گام‌هاي مثبت مانند در جستجوي اطلاعات و درمان، گام‌هاي منفي مانند انفصال و اختفاء) و در جستجوي حمايت (حمايت خانواده، حمايت همتايان، حمايت دوستان) بود. نتيجه‌گيري: بيماران در سازش با PCOS، در ابعاد مختلف زندگي فردي، خانوادگي و اجتماعي شرايط متفاوتي را تجربه مي‌كردند. عده‌اي در سازش مؤثر با اين شرايط موفق نبودند و برخي با حمايت ديگران و پشتوانه‌هاي اعتقادي و معنوي و روحيه مثبت به‌خوبي سازش يافته بودند. با‌توجه به شيوع بالاي مشكلات جسماني، رواني و اجتماعي در اين بيماران، تقويت منابع سازگاري و شناسايي موانع تطابق مي‌تواند در كمك به سازگاري اين گروه با بيماري و ارتقاي سطح كيفيت زندگي آنان مؤثر باشد.

Title: بررسی عوارض کاتترهای سابکلاوین و ژوگولار در بیماران دیالیزی

http://knh.shmu.ac.ir/index.php/site/article/view/393

مقدمه: جهت استفاده از وريدهاي بزرگ بدن، وريدهاي ساب كلاوين و ژوگولار بيشترين کاربرد را داشته و در اكثريت موارد از آنها استفاده مي‌شود. از آنجايي‌كه مزايا و معايب هر كدام از اين دو روش كاتترگذاري مشخص نيست، اين مطالعه جهت بررسي عوارض روش‌های كاتترگذاري ساب کلاوین و ژوگولار در بين بيماران دياليزي انجام شده تا روش بهتر را مشخص نمايد. مواد و روش‌ها: اين پژوهش به‌صورت كارآزمايي باليني تصادفي بر روي كليه بيماران دياليزي نيازمند به كاتتر وريدي كه در نيمه اول سال 90 به بيمارستان امام حسين (ع) مراجعه و رضايت شركت در طرح را داشته انجام شده است. در اين مطالعه بيماران به‌صورت تصادفي ساده به دو گروه مداخله (كاتتر ژوگولار) و گروه كنترل (كاتتر ساب کلاوین) تقسيم شده و پس از انجام اقدامات اوليه، در اتاق عمل به‌روش استاندارد براي آنها كاتتر وريدي گذاشته شد. سپس عوارض اين شانت‌گذاری مثل عفونت،‌ هموتوراكس، پنوموتوراكس 1، 6، 12، 24 و 48 ساعت بعد از عمل بررسي گرديد. در اين مطالعه از 136 بيمار مورد بررسي،‌ 72 بيمار (9/52%) در گروه مداخله و مابقي در گروه كنترل قرار گرفتند. نتايج: از نظر ميانگين بروز عفونت،‌ در گروه مداخله 3/5±9/6 بيمار و در گروه كنترل 1/6±3/7 بيمار دچار عفونت شدند كه تفاوت معناداري با هم نداشتند. درخصوص ميانگين بروز هموتوراكس در گروه مداخله 5/2 ± 2/4 بيمار و در گروه شاهد 1/4 ± 4/6 بيمار دچار اين عارضه شدند كه به‌طور معناداري در گروه كنترل بيشتر بود (003/0P<). همچنين درخصوص بروز عارضه پنوموتوراكس ميانگين بروز در گروه مداخله 7/6 ±7/10 بيمار و در گروه كنترل 2/7 ± 5/12 بيمار بود كه تفاوت معناداري بين آنها ديده نشد. همچنين اين مطالعه نشان داد سرعت عمل جراحي در گروه مداخله به‌طور معناداري از گروه كنترل بيشتر بوده است (05/0P<). نتيجه‌گيري: نتايج اين پژوهش نشان داد كه هر چند از هر دو روش كاتترگذاري ساب كلاوين و ژوگولار، باتوجه به شرايط بيمار و تجربه جراح مي‌توان استفاده كرد ولي روش ژوگولار به‌دليل عارضه كمتر هموتوراكس و سرعت عمل بيشتر مي‌تواند به‌عنوان روش ارجح مورد توجه قرار گيرد.

Title: تأثير تعدیل آلودگی‌های صوتی بر پاسخ‌های رفتاری نوزادان نارس بستری در بخش مراقبت‌های ویژه

http://knh.shmu.ac.ir/index.php/site/article/view/9

مقدمه: شواهد نشان مي‌دهد كه تولید صدا در بخش مراقبت‌های ویژه نوزادان نتايج نامطلوبي را در نوزادان نارس به‌دنبال خواهد داشت. مطالعه حاضر با هدف تعیین تأثير تعدیل آلودگی‌های صوتی در بخش مراقبت‌های ویژه بر معیارهای رفتاری نوزادان بستری در این بخش، انجام شده است. مواد و روش‌ها: نمونه‌های پژوهش شامل 110 نوزاد نارس بستری در بخش مراقبت‌های ویژه نوزادان بودند که در دو گروه 55 نفره تجربی و شاهد جای گرفتند. این نوزادان با ميانگين سن جنینی کمتر از 37 هفته و ميانگين وزن کمتر از 2500 گرم در زمان تولد به دنیا آمده، کمتر از 28 روز سن داشتند، حداقل 2 روز در بخش مراقبت‌های ویژه بستری بودند و تحت تهویه مکانیکی نیز قرار نداشتند. براي نمونه‌گيري در 2 مقطع زمانی انجام شد؛ در مقطع زمانی اول، هر نوزاد دارای معیارهای ورود به پژوهش در گروه شاهد قرار گرفته و در مقطع زمانی دوم، نوزادان واجد شرایط در گروه تجربی قرار گرفتند. در مقطع زمانی اول، پرستار آموزش دیده با استفاده از دستگاه صداسنج، تراز فشار صوت در شبکه وزنی A (صدای منطبق با شنوایی انسان) و C (صدای منطبق با صدای موجود در محیط) و تراز معادل فشار صوت LAeq و معیارهای رفتاری نوزادان را سنجیده است. در مقطع زمانی دوم، مداخلات تعدیل‌کننده آلودگی‌های صوتی به‌مدت 6 هفته در گروه مداخله اجرا و سپس به‌روش قبلي، تراز فشار صوت در شبکه وزنی A و C و تراز معادل فشار صوت LAeq و همچنین معیارهای رفتاری نوزادان سنجش گرديد. نتایج: تفاوت آماری معناداری در میانگین پاسخ‌های رفتاری نوزادان دو گروه مشاهده نشد؛ به‌علاوه تراز فشار صوت در شبکه وزنی C و A در گروه تجربي به شکل معتاداری کمتر از گروه شاهد بود. همچنين تفاوت آماری معناداری در تراز معادل فشار صوت در شبکه بین دو گروه مشاهده نشد. نتیجه‌گیری: این مطالعه نشان داد که در بخش‌های مراقبت ویژه نوزادان مورد مطالعه، میزان آلودگی صوتی بیش از حد استاندارد می‌باشد. ليكن براي نشان دادن تأثير آن بر پاسخ‌های رفتاری نوزادان پژوهش‌های بیشتري در این زمینه بايد انجام پذيرد.

Title: بررسی تأثیر آموزش بر مبنای "مدل تلفیقی رفتار برنامه‌ریزی شده و خودکارآمدی" بر انجام رفتارهای ارتقاء‌دهنده سلامت زنان کارگر در سنین باروری

http://knh.shmu.ac.ir/index.php/site/article/view/357

مقدمه: مدل رفتار برنامه‌ریزی شده چارچوب مفیدی برای پیش‌بینی رفتارهاي بهداشتی فراهم می‌نماید. اضافه نمودن خودکارآمدی به مدل رفتار برنامه‌ریزی شده، عامل مؤثرتري در پیش‌بینی رفتار مي‌باشد. لذا این مطالعه با هدف بررسی تأثیر مداخله آموزشی بر مبنای " مدل تلفیقی رفتار برنامه‌ریزی شده و خودکارآمدی" بر رفتارهای ارتقاء‌دهنده سلامت زنان کارگر سنین باروری صورت پذیرفت. مواد و روش‌ها: این پژوهش یک مطالعه تجربی بود. تعداد 70 نفر از زنان کارگر در دو کارخانه لواشک‌سازی (35 نفر گروه مداخله و 35 نفر گروه کنترل) به‌طور تصادفی انتخاب شدند. پرسشنامه معتبر و پایای"بررسی رفتارهای ارتقاء‌دهنده سلامت زنان کارگر"؛ ابزار جمع‌آوری داده‌ها بود. ابزار یک بار قبل و یک با بعد از مداخله آموزشی تکمیل گردید. داده‌ها توسط آمار توصیفی و استنباطی مورد تجزیه‌و‌تحلیل قرار گرفت. نتايج: نتایج پیش آزمون نشان داد قبل از مداخله آموزشی در تمامی حیطه‌ها (نگرش، هنجار فردی، درک کنترل رفتار، خودكارآمدی، قصد و رفتار) بین گروه مداخله و کنترل تفاوت معنی‌داری وجود نداشت (05/0≤P) اما بعد از مداخله آموزشی میانگین امتیازات نگرش، هنجار فردی، درک کنترل رفتار، خودکارآمدی، قصد و رفتار نسبت‌به رفتارهای ارتقاء‌دهنده سلامت در گروه مداخله افزایش یافت. همچنین بیشترین تفاوت میانگین در حیطه خودکارآمدی بود. نتیجه‌گیری: "مدل تلفیقی رفتار برنامه‌ریزی شده و خودکارآمدی" به‌عنوان الگوی قابل قبولی جهت طراحی مداخلات و ارتقای رفتارهای ارتقاء‌دهنده سلامت زنان کارگر پیشنهاد می‌گردد.

Title: بررسی اثر عصاره بیضه در مقایسه با عوامل رشد اختصاصی بر روی حفظ خود نوزایی سلول‌های بنیادی اسپرماتوگونی

http://knh.shmu.ac.ir/index.php/site/article/view/10

مقدمه: سلول‌های بنیادی اسپرماتوگونی به‌عنوان سلول بنیادی اختصاصی بافت بیضه وظیفه تولید مداوم اسپرم را در جنس نر بر عهده دارند. حفظ خودنوزایی این سلول‌ها جهت تضمین اسپرم‌زایی مداوم ضروری بوده و کنام بیضه با فراهم‌آوردن شرایط مناسب این مهم را برعهده دارد. تا‌کنون در شرایط آزمایشگاهی مواد و عوامل رشد اختصاصی جهت جایگزینی این کنام معرفی شده‌اند که اغلب این مطالعات بر روی سلول‌های بنیادی اسپرماتوگونی پستانداران صورت گرفته است. لذا در این مطالعه اثر عصاره بیضه موش و خروس بالغ در مقایسه با عوامل رشد بر روی ویژگی کلنی‌زایی سلول‌های بنیادی اسپرماتوگونی خروس مورد بررسي قرار گرفت. مواد و روش‌ها در این مطالعه اثر عصاره بیضه موش و خروس بالغ در مقایسه با سه عامل رشد GDNF، bFGF و LIF بر روی ویژگی کلنی‌زایی سلول‌های بنیادی اسپرماتوگونی خروس در طی چهار روز تیمار تحت بررسي قرار گرفت. پس از مشخص شدن غلظت بهینه عوامل مورد آزمایش، سلول‌ها به‌مدت دوهفته تحت تیمار قرار گرفتند و سپس بررسی بیان ژن OCT4 به‌عنوان یک ژن حائز اهمیت در سلول‌های بنیادی اسپرماتوگونی با روش Real-time RT-PCR انجام پذیرفت. نتایج: نتایج بررسی کلنی‌زایی نشان داد که پس از گذشت چهار روز از تیمار با عصاره بیضه موش و خروس بالغ در مقایسه با گروه كنترل و همچنین عوامل رشد GDNF، bFGF و LIF در مقایسه با نمونه‌هاي كنترل تفاوت قابل ملاحظه‌ای را در تعداد کلنی‌های تشکیل شده نشان دادند. نتایج بررسی بیان ژني نيز نشان داد كه پس از گذشت دو هفته از تیمار، بيان ژن OCT4 به‌طور معني‌داري افزايش مي‌يابد. نتیجه‌گیری: نتایج این مطالعه نشان داد عصاره بیضه می‌تواند به‌عنوان جایگزینی مناسب اما قابل ارتقا برای عوامل رشد گران قیمت مطرح گردد.

Title: اثر حفاظتی آگماتین در برابر اختلال حافظه ناشی از تزريق استرپتوزوتوسین داخل بطنی در موش‌هاي صحرایی نر

http://knh.shmu.ac.ir/index.php/site/article/view/270

مقدمه: تجویز استرپتوزوتوسین به‌صورت داخل بطنی موجب اختلال یادگیری و حافظه می‌شود. مکانیسم‌های مختلفی مانند استرس اکسیداتیو و اختلال سیگنالینگ انسولین برای این اختلال حافظه مطرح شده است. با‌توجه به اینکه تغییرات فوق در بیماری آلزایمر نیز رخ می‌دهد این مدل جهت بررسی بیماری آلزایمر به‌صورت وسیعی استفاده می‌شود. آگماتین پلی‌آمینی است که از دکربوکسیلاسیون ال- آرژینین مشتق می‌شود. اخيراً نقش حفاظت‌گر نوروني براي اين شناسايي شده است. لذا در اين مطالعه اثر حفاظتی آگماتین در برابر اختلال حافظه ناشی از استرپتوزوتوسین مورد بررسي قرار گرفت. مواد و روش‌ها: رت‌های نر از نژاد Sprague Dawley در محدوده وزنی 200 تا 250 گرم در این مطالعه استفاده شدند. ابتدا کانول‌هایی به‌صورت دو طرفه در بطن جانبی جای‌گذاری شد. سپس استرپتوزوتوسین در روزهای 1 و 3 با دوزmg/kg  3 در دو دوز منقسم تزریق گرديد. تزریق درون صفاقی آگماتین نيز با دوزهای mg/kg 40 و 80 از روز 4 تا روز 14 به‌صورت یک در میان انجام شد. همچنين توانایی یادگیری و حافظه در روز 15 تا 18 به‌وسیله ماز آبی موریس ارزیابی شد. رت‌ها در 3 روز اول آموزش دیدند و در روز جهارم آزمون پروب انجام شد. همچنين به‌منظور بررسی اثر داروها بر انگیزش یا هماهنگی حسی حرکتی، پس از آزمون، آزمون سكوي آشکار نيز انجام شد. نتایج: نتایج نشان دادند که آگماتین با دوز mg/kg 80 و نهmg/kg 40 از اختلال یادگیری و حافظه ناشي از تزريق داخل بطني استرپتوزوتوسین در ماز آبی موریس جلوگیری می‌کند. نتیجه‌گیری: به‌نظر می‌رسد که آگماتین می‌تواند در اختلالات يادگيري و حافظه ناشی ازجمله در بیماری آلزایمر سودمند باشد.

Title: كاهش ميزان گلوتاتيون بافت كبد به‌دنبال ايسكمي– پرفيوژن مجدد كليوي

http://knh.shmu.ac.ir/index.php/site/article/view/325

مقدمه: ایسکمی- پرفیوژن مجدد (IR) کلیوی يكي از علل شايع آسيب حاد كليوي مي‌باشد. اين شرايط سبب اختلال در عملکرد اندام‌هاي دوردست (remote organ) مانند ریه، قلب و کبد نيز می‌گردد. لذا در این مطالعه اثر لکوسیت‌ها بر ميزان گلوتاتيون بافت كبد به‌دنبال آسیب ناشي از ایسکمی- پرفیوژن مجدد کلیوی، مورد بررسي قرار گرفت. مواد و روش‌ها: موش‌های سوری Inbred در دو گروه كنترل و IR کلیوی دوطرفه که 50 دقیقه ایسکمی و به‌دنبال آن 3 ساعت پرفیوژن مجدد را تحمل کردند، قرار گرفتند. سپس نمونه‌ي خون و نمونه‌ي کبد موش‌ها در شرايط بیهوشي جمع‌آوری گردید. لکوسیت‌ها از خون جدا شده و به دو گروه گيرنده منتقل شدند: به اين ترتيب كه موش‌های گروه "گيرنده كنترل" لکوسیت‌های گروه كنترل و موش‌های گروه "گيرنده IR" لکوسیت‌های گروه IR را دریافت نمودند. بعد از 24 ساعت نيز تمامي موش‌های گيرنده بیهوش گرديده و نمونه‌های خون و کبد آنها جمع‌آوری شد. نتايج: در گروه موش‌های گيرنده IR نسبت‌به گروه موش‌های گيرنده كنترل گلوتاتیون (GSH) به‌طور معنی‌دار کاهش یافت. نتيجه‌گيري: اين نتايج نشان داد که آسيب كبدي ناشي از انتقال لكوسيت‌ها به‌دنبال ايسكمي- پرفيوژن مجدد كليوي، احتمالاً به سبب القاي استرس اکسیداتیو در اين بافت انجام مي‌شود.
